# A review of β-amyloid neuroimaging in Alzheimer's disease

**DOI:** 10.3389/fnins.2014.00327

**Published:** 2014-10-31

**Authors:** Paul A. Adlard, Bob A. Tran, David I. Finkelstein, Patricia M. Desmond, Leigh A. Johnston, Ashley I. Bush, Gary F. Egan

**Affiliations:** ^1^Division of Mental Health, The Florey Institute of Neuroscience and Mental Health, University of MelbourneParkville, VIC, Australia; ^2^Department of Radiology, University of MelbourneParkville, VIC, Australia; ^3^Department of Radiology, The Royal Melbourne HospitalParkville, VIC, Australia; ^4^Department of Electrical and Electronic Engineering, University of MelbourneParkville, VIC, Australia; ^5^Monash Biomedical Imaging, Monash UniversityClayton, VIC, Australia; ^6^School of Psychology and Psychiatry, Monash UniversityClayton, VIC, Australia

**Keywords:** neuroimaging, Alzheimer's disease, MRI, PET, mouse models, CT, biomarkers

## Abstract

Alzheimer's disease (AD) is the most common cause of dementia worldwide. As advancing age is the greatest risk factor for developing AD, the number of those afflicted is expected to increase markedly with the aging of the world's population. The inability to definitively diagnose AD until autopsy remains an impediment to establishing effective targeted treatments. Neuroimaging has enabled *in vivo* visualization of pathological changes in the brain associated with the disease, providing a greater understanding of its pathophysiological development and progression. However, neuroimaging biomarkers do not yet offer clear advantages over current clinical diagnostic criteria for them to be accepted into routine clinical use. Nonetheless, current insights from neuroimaging combined with the elucidation of biochemical and molecular processes in AD are informing the ongoing development of new imaging techniques and their application. Much of this research has been greatly assisted by the availability of transgenic mouse models of AD. In this review we summarize the main efforts of neuroimaging in AD in humans and in mouse models, with a specific focus on β-amyloid, and discuss the potential of new applications and novel approaches.

## Introduction

Alzheimer's disease (AD) is the most common cause of dementia worldwide, accounting for 65–75% of all cases of dementia (Bianchetti and Trabucch, 2001; Brookmeyer et al., 2011). It is characterized by neurodegenerative changes that are associated with early deficits in memory, but inexorably progresses to affect other cognitive domains. With progression of the disease, the affected individual's ability to function is eroded until all independence is lost. The annual incidence of AD rises markedly with age, from 53 new cases per 1000 people aged 65 to 74, to 170 new cases per 1000 people aged 75–84, and 230 new cases per 1000 people aged over 85 (Alzheimers, 2011). Similarly its prevalence increases exponentially with age, in the United States rising from 2.32% in those aged 71–79 years to 29.60% in those aged over 90 (Brookmeyer et al., 2011). Approximately 5% of cases occur as an inheritable trait passed on in a Mendelian manner, wherein the onset of symptoms develop relatively early in life, leading these to be termed early onset AD (EOAD) (van Gool and Eikelenboom, 2000; Rocchi et al., 2003; Minati et al., 2009). In contrast, the vast majority of cases occur sporadically as late onset AD (LOAD) and are multi-factorial in etiology. However, the single greatest risk factor for LOAD is age. Consequently, with increasing longevity and the overall aging of the world's growing population, the number of people with dementia is projected to increase by 234%, from a currently estimated 24.3 million people to 81.1 million people by 2040 (Ferri et al., 2005). This presents immense health and economic challenges for both the present and the future.

A major impediment to the management of AD is the inability to definitively diagnose the disease until post-mortem examination. The initial diagnosis is presumptive, based upon clinical evaluation and neuropsychological testing and the fulfillment of certain criteria as laid out in the International Classification of Disease (10th Revision; ICD-10), the Diagnostic and Statistical Manual of Mental Disorders (Fourth Edition, Text Revision; DSM-IV-TR) and the National Institute of Neurological and Communicative Disorders and Stroke-Alzheimer's Disease and Related Disorders Association (NINCDS-ADRDA) (Honig and Mayeux, 2001; Morris, 2003; Minati et al., 2009). Application of these criteria yields a diagnostic accuracy of 80–90% when compared to the histopathological gold standard (Morris, 2003; Masters et al., 2006; Minati et al., 2009). However, a variety of disorders can lead to or mimic dementia or can co-exist with AD, thereby worsening its progression. While conditions such as stroke, brain tumors, normal pressure hydrocephalus, infections and vitamin deficiency can be readily excluded, other causes of dementia that must be differentiated from AD, including vascular dementia, frontotemporal dementia and dementia with Lewy bodies may present greater diagnostic challenges. Furthermore, it is now evident that patients who will develop AD may present as a subset of people with mild cognitive impairment (MCI). These patients have subjective or objective impairment in a single cognitive domain that does not impair their functional capacity. The recognition of MCI as a prodrome to AD and that neuropathology develops well in advance of any clinical symptoms gives greater impetus to developing diagnostic and prognostic biomarkers for AD.

Neuroimaging presents immense potential for developing reliable biomarkers that can be viewed in the living brain. Key neuropathological features on which the definitive diagnosis of AD relies, seen at post-mortem in the brains of AD patients, are general atrophy of the cortex, neuron and synapse loss, extracellular plaques composed of insoluble β-amyloid (Aβ) and intraneuronal neurofibrillary tangles (NFTs) consisting of hyperphosphorylated tau (Perl, 2010). Developments in imaging enable these features to be visualized, either directly or indirectly, providing important information on the disease processes *in vivo*. Early and subtle structural changes, evident as atrophy in vulnerable brain regions such as the hippocampus and entorhinal cortex, can be detected with high-resolution magnetic resonance imaging (MRI). These changes are reflected at the cellular level by alterations in metabolites detected by magnetic resonance spectroscopy (MRS). Further changes are evident with functional imaging using functional MRI (fMRI), single positron emission computed tomography (SPECT) and positron emission tomography (PET). Functional abnormalities are surrogates of synaptic dysfunction/ loss in early AD and of neuronal loss with disease progression.

However, the most significant development is the ability to image the amyloid plaques, whose deposition and accumulation are widely viewed as fundamental to the pathological cascade leading to AD. Neuroimaging of plaques in humans using amyloid-labeling PET tracers and in transgenic (Tg) animal models, particularly mouse models of AD, has greatly expanded the understanding of amyloid in the context of AD pathogenesis. Yet, the picture that is emerging is that significant plaque accumulation occurs prior to the earliest clinical symptoms and amyloid burden has reached a steady state by the time of clinically diagnosed AD. It may also be less important in terms of neurotoxicity than other soluble oligomeric species of Aβ. Understanding the true role of amyloid pathology in the pathogenesis of AD will provide meaningful insight into the search for effective targeted therapeutics for this disease, but it will rely as much on developing neuroimaging in animal AD models as the validation of techniques in humans.

In this review we will focus on Aβ, as it is currently the most widely studied neuroimaging biomarker for the diagnosis and monitoring of disease. Furthermore, whilst opinions are divided, a large proportion of the scientific community believe that amyloid is a primary mediator of disease pathogenesis in AD. We will briefly discuss the current understanding of the pathological mechanisms that provide the theoretical underpinnings for amyloid imaging, therein exploring the appropriateness of its use as a biomarker of AD. Finally we will examine the development amyloid imaging in Tg mouse models for application in pre-clinical and clinical research. In particular, we will attempt to highlight the technical challenges faced in validating amyloid imaging for these applications.

## Pathophysiology of Alzheimer's disease

### Central role of β-amyloid in Alzheimer's disease

Central to the pathogenesis of AD is the Aβ protein, produced from the cleavage of the amyloid precursor protein (APP). Over 80% of cases of EOAD are accounted for by mutations in either the gene for APP or one of two other proteins presenilin-1 (PS1) and presenilin-2 (PS2), which form part of the γ-secretase complex (Rocchi et al., 2003). These mutations lead to an accelerated production of Aβ that exceeds its clearance mechanisms and subsequently results in its accumulation in the brain. In contrast, LOAD develops from impaired clearance of Aβ due to various factors (Zetzsche et al., 2010).

Cleavage of APP occurs sequentially by the action of either α-secretase or β-secretase (BACE-1) and γ-secretase (Masters et al., 2006; Murphy and LeVine, 2010). Whilst the majority of APP is processed by α-secretase to result in non-amyloidogenic cleavage products (Masters et al., 2006; Murphy and LeVine, 2010), a proportion is cleaved by β-secretase and γ-secretase to result in the formation of Aβ peptides (Murphy and LeVine, 2010). The predominant forms of Aβ produced are Aβ_40_ and Aβ_42_, of which the former is more common. However, the latter has a greater propensity for aggregating into oligomers and fibrils and is believed to be the more toxic product (Ballard et al., 2011). Degradation of Aβ has been attributed to a number of mechanisms. Extracellular enzymatic degradation may be carried out by neprilysin and insulin degrading enzyme, while intracellular lysosomal degradation may also play a role (Murphy and LeVine, 2010). In addition, the activity of microglia may also play an important role in clearance of both soluble Aβ and its fibrillar aggregates (Agostinho et al., 2010; Lee and Landreth, 2010).

The mechanisms leading to Aβ aggregation are not fully understood but a number of factors may be involved. It is likely that protein folding plays a crucial role in amyloidogenesis, as proteins seek to attain a quaternary structure that has a minimum energy and hence most stable conformation (Bharadwaj et al., 2009). An alternative but stable “misfolded” state may make Aβ prone to aggregation and mutations associated with FAD may predispose to misfolding (Bharadwaj et al., 2009). Similar to prion diseases, this abnormal aggregation and precipitation could be the cause of amyloidosis in AD. Aβ aggregation may be a consequence of interactions with other factors such as the metals zinc and copper, which are dysregulated in the AD brain (Bush, 2008; Kawahara, 2010).

How Aβ aggregates exert their neurotoxic effects is still unclear. Amyloid has been shown to be directly toxic to neuronal cell cultures (Golde et al., 2006). Evidence suggests a number of pathways by which Aβ acts. Chief among these is oxidative stress and inflammation, which have an established role in the biology of aging, and calcium dyshomeostasis (Reddy, 2009; Kawahara, 2010). In AD, these pathological processes have the early consequences of synaptic dysfunction and culminate later in neuronal loss. Amyloid burden, however, correlates poorly with disease severity, suggesting a lesser role for insoluble Aβ fibrils, while soluble Aβ oligomers appear to play the major part in neurotoxicity (Minati et al., 2009).

### Mechanisms of amyloid-β pathogenesis

Evidence strongly suggests oxidative stress has an early and important role in the pathogenesis of AD (Maynard et al., 2005; Agostinho et al., 2010; Muller et al., 2010; Santos et al., 2010). Redox reactions occurring during normal cellular respiration produce reactive oxygen species (ROS) such as superoxide anion, hydroxyl radical and hydrogen peroxide. ROS interact with and alter cellular components, including nucleic acids, lipids and proteins, causing damage to important cellular structures and impairing their functions. Molecular mechanisms have evolved to minimize and repair oxidative damage but they may be overwhelmed, tipping the balance toward oxidative stress. The consequence of accumulated oxidative damage is a molecular cascade resulting in cellular dysfunction and ultimately, the triggering of pathways leading to cell death (Sultana et al., 2009).

In AD, accelerated oxidative stress is likely driven by Aβ pathology in a number of ways. One mechanism is by interactions with and the dysregulation of important biological metals. Another is by impairment of mitochondria, the organelles in which cellular respiration resides and the main source of endogenous ROS in cells (Casadesus et al., 2004; Agostinho et al., 2010). Increased APP in mitochondrial membranes may be one cause of mitochondrial dysfunction in neurons (Kawahara, 2010). Furthermore, Aβ may also disrupt mitochondrial membrane function by inserting into it as oligomers, creating calcium-permeable channels (Reddy, 2009; Kawahara, 2010). This affects the flux of calcium across the membrane with resultant mitochondrial structural and functional damage (Reddy, 2009). Aβ oligomers are also incorporated into neuronal membranes, leading to a wider dysregulation of calcium homeostasis in the neuron (Kawahara, 2010). Disruption of calcium homeostasis affects cellular function and can trigger apoptotic pathways of cellular death. Therefore, Aβ-induced calcium dyshomeostasis may act synergistically with oxidative stress to cause neuronal dysfunction and death.

Both the increasing presence of Aβ aggregates and the release of substances by dying neurons incite neuroinflammation, which is an early event in AD (Agostinho et al., 2010; Glass et al., 2010; Lee and Landreth, 2010). Inflammatory mediators such as cytokines and activated complement components are elevated in vulnerable brain regions in AD (Agostinho et al., 2010; Lee and Landreth, 2010). Furthermore, the presence of activated microglia and astrogliosis around senile plaques support the role of inflammation (Agostinho et al., 2010; Glass et al., 2010; Lee and Landreth, 2010). Microglia and astrocytes may have neuroprotective functions by their scavenging and clearance of Aβ aggregates (Agostinho et al., 2010; Lee and Landreth, 2010). However, they may also mediate neurotoxic effects by release of pro-inflammatory cytokines, chemokines, ROS and complement proteins (Lee and Landreth, 2010). Furthermore, APP mRNA is upregulated by IL-1, an inflammation cytokine, further promoting amyloidosis and reinforcing a self-perpetuating cycle (Rogers and Lahiri, 2004).

How Aβ pathology is related to tau pathology remains unclear. However, there is growing evidence to support the direct or indirect interaction of Aβ with tau to accelerate NFT formation. It is likely that Aβ pathology lies upstream of tau pathology, as genetic mutations leading to elevated Aβ levels or aggregation also leads to NFT formation while no reciprocal relationship with tau accumulation has been found (Blurton-Jones and Laferla, 2006). Phosphorylation of tau at various sites promotes their aggregation and NFT formation while Aβ is shown to activate several tau kinases, providing a straight forward mechanism for induction of NFT formation by Aβ (Blurton-Jones and Laferla, 2006; Mi and Johnson, 2006). Other suggested links are: Aβ-induced inflammation with resulting induction and enhancement of tau phosphorylation by inflammatory cytokines; Aβ-induced proteasomal impairment of tau degradation; and dysregulation of axonal transport with possible bidirectional effects, leading to increases in Aβ as well as tau (Blurton-Jones and Laferla, 2006).

Accumulation of Aβ and abnormal tau pathology exerts an effect on synapses, affecting their density and function and consequently the function of neuronal circuits within the brain (Selkoe, 2008; Arendt, 2009; Palop and Mucke, 2010). Histopathological examination of AD brain tissue from patients within 2–4 years after clinical onset of AD reveals significant reductions in post-synaptic dendritic spine density. These changes are most marked in the hippocampus and least prominent in the occipital cortex, exhibiting differences in the regional distribution of synaptic alterations that matches the pattern outlined in the Braak and Braak staging system (Arendt, 2009). Furthermore, this reduction in synaptic density is already evident in patients with MCI (Arendt, 2009). Aβ oligomers, both synthetic and naturally occurring, have been shown to mediate synaptic dysfunction, impairing long-term potentiation as well as enhancing long-term depression (Selkoe, 2008; Shankar et al., 2008; Sultana et al., 2009; Palop and Mucke, 2010). Studies have shown elevated Aβ decreases excitatory glutamatergic synaptic transmission by reductions in post-synaptic α-amino-3-hydroxy-5-methyl-4-isoxazolepropionic acid receptor (AMPA) receptors and *N*-methyl-D-aspartate (NMDA) receptors (Palop and Mucke, 2010). Furthermore, accumulation of abnormally phosphorylated tau species within synaptic terminals in AD brains and APP transgenic mice has been reported, suggesting a link between tau and synaptic pathology (Arendt, 2009). Synaptic dysfunction consequently leads to alterations in brain activity and connectivity, evident as regional alterations in activity on functional studies (Bokde et al., 2009).

In summary, these data support a primary role for Aβ in the pathogenesis of AD (although clearly there are other aspects that are also very important). As such, the ability to detect Aβ in the brain of living individuals has long been heralded as a key to allow the earliest detection of AD, with the subsequent benefit of expedited treatment and the hope of extending the number of disease free years. There are several important caveats to this however. Firstly, Aβ accumulates in the brains of normal individuals. In the earliest stages this appears to correlate with subjective cognitive complaints, although not with objective measurements of memory and executive function (Amariglio et al., 2012). This may, however, represent an early indicator for the subsequent development of AD. The time frame between the first detection of Aβ by neuroimaging and the clinical manifestation of AD may also be quite long, as Aβ accumulates at quite a slow rate (Villemagne et al., 2013). Furthermore, whilst Aβ generally increases continuously from the levels found in younger healthy controls to those found in AD, it does not do so in a linear fashion, with rates of deposition slowing in more advanced stages of disease (Villemagne et al., 2013). That being said, understanding the natural history of Aβ formation and deposition will provide a greater understanding of the overall dynamics of the potential progression from healthy control, through MCI (individuals in this group have a transition rate to AD of approximately 30.5% Ellis et al., 2014) and into AD. This will ultimately provide a better prognostic indication for the individual with a given Aβ burden. In addition, such data will facilitate better controlled clinical studies by allowing Aß burden to be used as an intake/cutoff criteria for inclusion in drug studies.

Another important caveat is in regards to whether the level of Aβ accumulation, as observed through neuroimaging methodologies, is a good correlate of cognitive function—this remains a contentious issue, with the majority of studies suggesting that it is a poor marker of disease severity. However, a recent study demonstrated, for the first time, that there was an association between the rate of Aβ deposition and memory decline in a cohort of AD patients (Villemagne et al., 2013). These findings arose out of the Australian Imaging, Biomarkers and Lifestyle study, which is a multi-year prospective study of 1112 individuals with either AD, MCI or no pathology (age-matched healthy controls), and perhaps demonstrates the need for such large, well-controlled studies in order to discern correlations between Aβ burden and cognitive function. Thus, there are several important outcomes, both for the individual and for the community, that serve as important rationales for continuing neuroimaging initiatives in AD.

## Neuroimaging in Alzheimer's disease

The current clinical role of neuroimaging in AD is the exclusion of any other disease pathology that may be causative of cognitive decline (Ferreira and Busatto, 2011; McKhann et al., 2011). These include cerebrovascular diseases, tumor, subdural haematoma or other causes of dementia, including dementia with Lewy bodies and frontotemporal dementia (Lee et al., 2003; Small et al., 2008; Mistur et al., 2009). Additionally, imaging may reveal cerebral atrophy evidenced by ventricular and sulcal enlargement, non-specific signs which can only support the diagnosis of AD as they are also observed in normal aging and other dementias. The two imaging modalities in routine use are computed tomography (CT) and MRI. Of the two, MRI is preferred due to its far superior soft tissue resolution and hence its ability to provide detailed structural information (Ferreira and Busatto, 2011). Nonetheless, CT has limited utility in the diagnostic work up, particularly where MRI is contraindicated (Ferreira and Busatto, 2011).

Recently, with advances in imaging technology, studies using MRI and PET neuroimaging of AD have identified a number of potential biomarkers (Ferreira and Busatto, 2011). However, these biomarkers have yet to gain acceptance as a diagnostic criteria (McKhann et al., 2011). Consequently their main application is in the area of research, with the aim of furthering understanding into the pathological processes of AD and also of improving their reliability for routine clinical use. With continuing advances, the potential of imaging biomarkers draws closer to realization.

### Magnetic resonance imaging

The high resolution provided by MRI has made it especially useful in studying the morphological details of the AD brain *in situ*. Analyses in quantitative structural studies of brain atrophy are commonly applied to gray matter changes in T_1_-weighted images and have been either cross-sectional or longitudinal (Vemuri and Jack, 2010). Cross-sectional volumetric analysis of medial temporal lobe (MTL) structures can be done qualitatively by visual assessment or quantitatively by analysis of regions of interest either using manual tracing and measurement or automated techniques (Vemuri and Jack, 2010). Voxel-based analysis has also been applied for comparisons between an AD group and a control group to detect subtle differences (Glodzik-Sobanska et al., 2005; Vemuri and Jack, 2010). Longitudinal methods of analysis include global atrophy quantification by analysis of brain boundary shifts, and tensor-based morphometry, a 3-dimensional voxel-based method (Glodzik-Sobanska et al., 2005; Coimbra et al., 2006; Vemuri and Jack, 2010). While brain atrophy is not specific to AD, studies have revealed atrophy of the MTL is well correlated with changes in cognition and disease progression (Coimbra et al., 2006; Frisoni et al., 2010; Vemuri and Jack, 2010). In particular, atrophy of the hippocampus and entorhinal cortex is of particular value as a biomarker due to their involvement in the earliest stage of disease and strong correlation with NFT pathology (Glodzik-Sobanska et al., 2005; Ramani et al., 2006; Vemuri and Jack, 2010). Hippocampal volume is reduced in MCI patients by 10–15% while in mild and moderate AD it is reduced by 15–30% and 30–40% respectively (Lehericy et al., 2007; Frisoni et al., 2010). The rate of atrophy is also predictive of progression, being 4–6% per year in MCI/AD patients compared to 1–2% in age-matched controls (Coimbra et al., 2006). As will be discussed later, MR imaging has been successfully applied to animal models of AD, and in some cases has provided unique observations of potential relevance to the human condition. Volumetric losses present outside of forebrain structures, for example, have been reported to predict sites of future amyloid formation (Badea et al., 2010).

Structural studies have also utilized other MR imaging techniques to image pathological alterations in both gray and white matter. Diffusion weighted imaging (DWI) with apparent diffusion coefficient (ADC) mapping and diffusion tensor imaging (DTI) has been used to investigate the white matter changes in areas such as the temporal lobe, hippocampus and corpus callosum (Ramani et al., 2006; Small et al., 2008; Stebbins and Murphy, 2009). In particular, DTI is capable of assessing microstructural and connective changes in the hippocampus and its related structures, arising from neuronal loss and axonal degeneration (Yakushev et al., 2011a,b; Hattori et al., 2012). Alternatively, magnetization transfer imaging (MTI) is able to detect structural damage as a reduction in the magnetization transfer rate (MTR) reflects decreased tissue homogeneity from pathological changes such as neuronal loss and gliosis in gray matter, and demyelination and axonal loss in white matter (Glodzik-Sobanska et al., 2005; Ramani et al., 2006). Decreased MTR is found in both white and gray matter in AD patients and has been reported in the hippocampus in very mild AD (Ramani et al., 2006).

At a molecular level, cellular changes associated with neuronal pathology can be studied using MRS. The most commonly studied metabolites in MRS experiments of the brains of AD patients are N-acetyl-aspartate (NAA), choline and creatinine, which are associated with neuronal function, membrane turnover and energy consumption respectively (Lee et al., 2003; Coimbra et al., 2006; Scott et al., 2011). NAA has been found to be decreased in AD, consistent with neuronal damage (Coimbra et al., 2006). As the levels of choline or creatinine are relatively unchanged, the ratio of NAA to these metabolites may be a useful biomarker for AD (Lee et al., 2003; Coimbra et al., 2006). Other metabolites are also being currently investigated for their value in early diagnosis. Examples include myo-inositol (mIns), which may be a marker of glial activation and hence neuroinflammation, lactate for monitoring anaerobic metabolism indicative of hypoxia and ischaemia, and glutamine/glutamate for evaluating regulation of neurotransmission (Ashford et al., 2011).

While neuronal loss is an obvious endpoint resulting from the progression of AD pathology, it is now evident that this is preceded by neuronal dysfunction. This can be measured by changes in cellular metabolic rate, which on a regional level can be visualized *in vivo* as changes in the metabolic demand reflected by decreased vascular perfusion and oxygen consumption (Wu and Small, 2006). Consequently there are a number of functional MRI (fMRI) studies that have assessed the consequence of neuropathology in terms of neuronal activity and connectivity. These studies are based either on changes in cerebral blood flow and cerebral blood volume or changes revealed by the blood oxygen level dependent (BOLD) effect (Wu and Small, 2006; Brickman et al., 2009; Beckmann, 2011). Estimation of changes in cerebral blood flow and cerebral blood volume involves either the injection of intravenous paramagnetic contrast, such as gadolinium or superparamagnetic iron oxide nanoparticles or the technique of arterial spin labeling (ASL) (Wu and Small, 2006; Brickman et al., 2009; Beckmann, 2011). In contrast, the BOLD effect utilizes the paramagnetic properties of deoxyhaemoglobin. Activation therefore increases CBF out of keeping to metabolic needs. This leads to an increase in oxyhaemoglobin and a relative decrease in deoxyhaemoglobin which leads to a relative increase in signal on T2^*^ weighted imaging (Wu and Small, 2006; Brickman et al., 2009; Beckmann, 2011). FMRI studies have identified abnormalities in activity associated with a variety of cognitive tasks in a number of regions (Dickerson and Sperling, 2009; Sperling et al., 2010). Compared to healthy older controls, AD patients are reported to exhibit decreased hippocampal and parahippocampus activity, as well as decreased activity in the frontal and prefrontal regions (Sperling et al., 2010). Also, AD patients show less coordinated activity in the default mode network (DMN), a functional network consisting of the posterior cingulate, hippocampus, lateral inferior parietal and medial frontal lobes, that is most active at rest and reduces in activity during the performance of cognitive tasks (Small et al., 2008; Bokde et al., 2009; Dickerson and Sperling, 2009; Sperling et al., 2010).

Though imaging functional and structural consequences has provided many insights into AD, it is the potential to visualize molecular pathology that has stirred the imagination in MRI research. Since the first observations by Benveniste et al. (1999) that hypointensities on T^*^_2_-weighted MRI of *ex vivo* AD brains corresponded to neuritic plaques there has been great interest in the use of MRI in AD. Despite contrary findings by Dhenain et al. (2002), research into the development of MRI techniques to improve visualization and quantification of Aβ deposits *in vivo* has been ongoing. However, much of the imaging of human AD brains has been done *ex vivo* (Benveniste et al., 1999; Dhenain et al., 2002; House et al., 2007, 2008; Schrag et al., 2010; Nabuurs et al., 2011). Analyses of amyloid plaques suggest their inherent contrast on T^*^_2_-weighted imaging is attributable to increased iron content relative to surrounding tissues (Benveniste et al., 1999; Nakada et al., 2008; Nabuurs et al., 2011). Similar to other neurodegenerative diseases such as Parkinson's disease, iron dyshomeostasis has an important and early role in the disease process (Bartzokis et al., 2004). Abnormal Fe accumulation in the AD brain is evident from MR measurements of transverse relation rates (R_2_) in post-mortem studies (House et al., 2007, 2008). However, while these studies found a correlation between iron and R_2_, amyloid plaque density was not correlated with iron concentrations. Nonetheless, plaque-associated iron can potentially be harnessed to enhance their contrast by susceptibility-weighted imaging (SWI) in living patients (Nakada et al., 2008).

Further work is still needed to validate the clinical use of *in vivo* functional and plaque MRI in living patients with AD. For plaque imaging, one need is for improvement in the time efficiency of the scanning while achieving the required contrast and resolution. Another problem lies in the difficulty in correlating this imaging with histology. This is partly due to artifacts arising from other sources of iron (blood vessels, micro-hemorrhages) or as a result of head motion during long scanning times (Versluis et al., 2010). Because of this, there have been very few *in vivo* studies in living subjects (Nakada et al., 2008; Versluis et al., 2010). There are also limitations related to functional imaging with ASL and BOLD. Firstly, there is potential bias from the use of external stimuli and differences in cognitive task performance, which requires carefully constructed experimental paradigms for study validity. Secondly, high temporal resolution in the order of seconds is needed to capture transient evoked changes, making this technique very sensitive to head motion and compromising spatial resolution. Thirdly, disease related changes to brain structure make changes in responses difficult to interpret. Finally, fMRI produces relative rather than quantitative measures of brain activity, which can be problematic when applied to diagnostic or longitudinal monitoring purposes (Brickman et al., 2009; Dickerson and Sperling, 2009).

### Radionuclide imaging

Early radionuclide imaging in AD made use of PET and SPECT to evaluate functional alterations in the brain (Mosconi et al., 2010). SPECT analysis using Tc-99-labeled hexamethylpropyleneamine oxime (Tc-99-HMPAO) is able to show a global reduction in cerebral blood flow along with focal changes in terms of temporoparietal hypoperfusion (Lee et al., 2003; Coimbra et al., 2006). However, the predictive accuracy of SPECT for diagnosing AD is variable but generally found to be less than PET, making it of limited clinical use (Lee et al., 2003; Coimbra et al., 2006). The most common PET tracer used in functional studies is 2-[^18^Flfluoro-2-Deoxy-D-glucose (FDG). FGD PET studies show a specific regional pattern of cerebral glucose metabolism in AD compared to controls. Deficits are consistently observed in the temporoparietal, MTL and posterior cingulate cortices, and later in the frontal cortex as the disease progresses (Lee et al., 2003; Coimbra et al., 2006; Mosconi et al., 2010). Notably the primary sensory, motor and visual cortices, cerebellum, striatum and basal ganglia are preserved (Lee et al., 2003; Mosconi et al., 2010). This pattern can be used to distinguish AD from other forms of dementia such as FTD and DWLB, and from cerebrovascular disease (Mosconi et al., 2010). Evidence also suggests that these metabolic reductions precede clinical symptoms as well as structural brain changes in predisposed people (Mistur et al., 2009; Mosconi et al., 2010). Thus, it is suggested that FDG PET may be useful in AD prognostication and as an adjunct to clinical assessment for diagnosis (Lee et al., 2003; Mistur et al., 2009; Mosconi et al., 2010).

More recently the development of peptide and antibody probes has extended the use of PET beyond functional analysis to allow direct imaging of amyloid *in vivo*. Many of the molecular probes are derivatives of histological dyes such as Congo Red, thioflavin S and T, Acridine Orange and Chrysamine-G (Coimbra et al., 2006; Fodero-Tavoletti et al., 2009) or other molecules such as styrylbenzene. Two of the most extensively used ligands are [^11^C]2-(4′-methylaminophenyl)-6-hydroxybenzothiazole (Pittsburgh Compound B or PIB) (Small et al., 2008; Fodero-Tavoletti et al., 2009; Mosconi et al., 2010) and 2-(1-{6-[2-[^18^F]-fluoroethyl)(methyl)amino]-2-naphthyl}ethylidene) malononitrile ([^18^F]-FDDNP) (Small et al., 2008; Fodero-Tavoletti et al., 2009; Mosconi et al., 2010). [^18^F]-FDDNP was the first ligand successfully employed in humans (Rabinovici and Jagust, 2009). However, it binds both diffuse and dense-core amyloid along with tau (Coimbra et al., 2006; Mistur et al., 2009; Rabinovici and Jagust, 2009), complicating its interpretation and limiting its ability to differentiate AD from FTD and other tauopathies.

PIB on the other hand binds with high affinity and specificity to fibrillar Aβ (Coimbra et al., 2006; Fodero-Tavoletti et al., 2009; Mosconi et al., 2010) and its *in vivo* PET signal correlates strongly with *in vitro* measures of Aβ burden in post-mortem AD brains (Rabinovici and Jagust, 2009). Studies have demonstrated elevated retention of PIB in the brains of AD patients compared to healthy controls (Coimbra et al., 2006; Small et al., 2008; Mosconi et al., 2010). Significant PIB retention is found in 90% of clinically diagnosed AD patients and approximately 60% of individuals with MCI and 25–30% of normal elderly (Fodero-Tavoletti et al., 2009; Mosconi et al., 2010). The regional distribution of high retention in AD brains is in areas known to have high amyloid deposition, most prominently the frontal cortex, parietal cortex, lateral temporal cortex, posterior cingulated cortex/precuneus, thalamus and striatum (Coimbra et al., 2006; Rabinovici and Jagust, 2009; Mosconi et al., 2010). This pattern of distribution allows differentiation of AD from FTD and Parkinson's disease (Mosconi et al., 2010), though significant retention and a similar pattern can also seen in DWLB (Fodero-Tavoletti et al., 2009; Mosconi et al., 2010; Villemagne and Rowe, 2011). Imaging with PIB has also provided important insights into the relationship between amyloid burden and various markers of AD, such as cognitive decline (Rowe et al., 2010), functional alterations (Hedden et al., 2009; Rabinovici and Jagust, 2009; Shin et al., 2010, 2011; Mormino et al., 2012) and regional brain atrophy (Jack et al., 2008; Chetelat et al., 2010; Rowe et al., 2010).

A distinct disadvantage of PIB imaging is the short half-life of ^11^C, which is around 20 min. This necessitates the existence of a cyclotron in close proximity for production of the radioisotope label. Labeling of PET imaging probes with ^18^F, a radioisotope with a half-life of 110 min removes this limitation (Mistur et al., 2009). This approach has yielded a number of potential radiotracers, such as the 3′-fluoro-derivative of PIB, [^18^F]-3′-FPIB (flutemetamol), and the strylpyridine and stilbene derivatives [^18^F]-AV-45 (florbetapir) and [^18^F]-AV-1 (florbetaben) (Herholz and Ebmeier, 2011; Vallabhajosula, 2011). Not surprisingly, [^18^F]-3′-FPIB has near identical binding properties to PIB and performs similarly in clinical studies (Vallabhajosula, 2011). [^18^F]-AV-45 and [^18^F]-AV-1 also exhibit high affinity binding to fibrillar amyloid similar to PIB (Herholz and Ebmeier, 2011; Vallabhajosula, 2011). Ongoing development in amyloid-specific PET ligands will improve their utility and widen their accessibility for both research purposes and potentially routine clinical applications.

## Advances from pre-clinical neuroimaging in mouse models

### Models of Alzheimer's disease

Animal models of AD have been extensively utilized in the investigation of the disease. The best model is the aged monkey, such as the Caribbean vervet monkey, lemur, cotton-top tamarin, rhesus monkey and squirrel monkey (Philipson et al., 2010). However, the time and cost involved makes primate models prohibitive for routine use in research (Crews et al., 2010). There have been a variety of other models including: rats, rabbits, dogs, the fruit fly *Drosophila melanogaster*, the nematode *Caenorhabditis elegans* and two types of fish, the sea lamprey, *Petromyzon marinus* and the zebrafish (Götz et al., 2004; Götz and Götz, 2009; Crews et al., 2010; Murphy and LeVine, 2010; Philipson et al., 2010). However, the overwhelming majority of research has been carried out in transgenic mouse models, which confer advantages in terms of time and cost-effectiveness. At the same time they allow the detailed analysis of pathological mechanisms at molecular, anatomical and cognitive/behavioral levels.

Early models expressing human Aβ fragments, wild type human APP, or fusion proteins containing the C-terminal fragment of wild type APP or Aβ_42_ showed little or sparse AD-like neuropathology or atypical Aβ deposition (Philipson et al., 2010). However, with identification of EOAD-associated APP mutations, transgenic mouse models were developed expressing high levels of human APP incorporating these mutations. The first APP transgenic mouse was the PDAPP mouse, which incorporated one of the earliest APP mutations identified, the Indiana mutation (V717F) (Philipson et al., 2010; Wisniewski and Sigurdsson, 2010). PDAPP mice develop both senile and diffuse plaques from 9 months of age and showed deficits in learning and memory (Elder et al., 2010; Philipson et al., 2010). This was followed by what is the most commonly used APP transgenic model, the Tg2576 mouse harboring human APP with the Swedish mutation (KM670/671NL) under the control of the prion promoter. These mice have five-fold overexpression of human APP, develop Aβ deposits from 9 to 12 months of age and exhibit substantial cerebral amyloid angiopathy (CAA) (Crews et al., 2010; Philipson et al., 2010; Wisniewski and Sigurdsson, 2010). Other engineered models have incorporated other mutations such as the London (V717I), Arctic (E693G), Iowa (D694N), Flemish (A692G), or Dutch (E693Q) mutation, resulting in various patterns of Aβ deposition and pathology. For example, mice expressing the Dutch and Iowa mutations have a propensity for CAA and diffuse Aβ deposits. However, in most of these transgenic mice there is a lack of neuronal loss. This lead to the development of models expressing combinations of different APP mutations in an attempt to enhance neuronal toxicity, such as the APP23 mouse which harbors both the Swedish and London mutations (Crews et al., 2010; Elder et al., 2010; Philipson et al., 2010; Wisniewski and Sigurdsson, 2010).

Following the identification of presenilin mutations, these were similarly incorporated into mouse models. However, singly transgenic mice with either PS1 or PS2 did not develop plaques despite consistently showing elevated levels of Aβ_42_. Subsequent cross breeding of APP and PS transgenic mice, as well as introduction of knockin PS1 mutations in APP mutated mice produced doubly transgenic models. Different combinations of APP mutations with PS1 or PS2 mutations have produced a variety of APP/PS1 double transgenic models. One example is the PSAPP model, produced by crossing PS1 M146L or M146V mutant mice with Tg2576 mice. These mice showed markedly accelerated amyloid deposition from an earlier age and increased overall plaque numbers, as well as early cognitive deficits (Crews et al., 2010; Elder et al., 2010; Philipson et al., 2010).

These models of AD have been used to explore various aspects of its pathophysiology, ranging from Aβ metabolism and amyloid deposition to the effect of APOE genotype as well as tau and NFT pathology (Götz et al., 2004). However, while the amyloid deposits in mouse models closely resemble human amyloid, not all aspects of AD pathology are reproduced in transgenic mice. Except for triple transgenic mice, which incorporate tau mutation, the mice lack NFTs. Another difference is that amyloid deposition is not associated with significant neuronal loss, apart from when multiple mutations in either or both of APP and PS1 in double transgenic mice were introduced (Golde et al., 2006; Fiala, 2007; Elder et al., 2010).

Despite these limitations mouse models play an important role in ongoing research in AD, providing valuable insights into pathophysiology. Furthermore, through the knowledge gained these models are facilitating the search for new therapies and improved methods for diagnosis.

### Magnetic resonance imaging in mouse models

Research into the imaging of AD pathology in transgenic AD mouse models using MR has followed two general strategies. One is the indirect measure of AD pathology reflected in changes in relaxometry. The other is to develop techniques to utilize or enhance the inherent contrast of amyloid plaques so as to directly visualize them. Relaxometry studies involve statistical analysis of measures of longitudinal and transverse relaxation values in regions of interest for differences between AD transgenic mouse brains and age-matched non-transgenic mouse brains that could be attributed to Aβ deposition. The advantage of this method is, being based on ROIs, it is less reliant on resolution (Muskulus et al., 2009). In direct imaging of plaques the very small size of many plaques requires a resolution with appropriately small voxel dimensions. This increases scanning time and reduces the signal-to-noise ratio, impeding the routine application of MR amyloid imaging in *in vivo* research. Furthermore, the mechanisms underpinning signal changes are not yet fully understood (Braakman et al., 2009; Chamberlain et al., 2011). Consequently there is a great deal of ongoing research effort into methods for improving contrast and resolution in MR imaging and correlating this imaging to the biophysics and physiology of AD pathology.

Helpern et al. (2004) were the first to acquire parametric maps of T_2_ and T_1_ relaxation times and proton density to analyse changes in measured values that may reflect altered cellular physiology secondary to AD pathology (refer to Table 1). Of these three measures they found only T_2_ was reduced in the hippocampus, cingulate and retrosplenial cortex of APP/PS1 mice compared to PS1 and non-Tg mice, while T_1_ and proton density were unchanged between the genotypes. The correlation between reduction in T_2_ relaxation time and increased amyloid burden was later confirmed by other studies (Falangola et al., 2005a; Vanhoutte et al., 2005; Borthakur et al., 2006; Braakman et al., 2006; El Tannir El Tayara et al., 2006; El Tayara Nel et al., 2007). Apart from confirming T_2_ changes in APP/PS1 mice, Falangola et al. (2005b) applied an algorithm to register mapped T_2_ values to anatomical regions for comparison of differences between groups of 18 months old APP/PS mice, PS mice and non-Tg mice, as well as between groups of 6 weeks old and 18 months old PS and non-Tg mice. Helpern et al. (2004) attributed the T_2_ reduction to the higher amyloid plaque burden in these areas. However, this was inferred from the similarity of the pattern of hypointensities demonstrated by *ex vivo* MR imaging in APP/PS1 mice to the distribution of plaques in histology described in the literature.

**Table 1:** **Summary of MRI studies utilizing indirect measures of AD pathology in transgenic mouse models**.

**Study**	**Protocol/s**	**Field**	**Model/s**	**Study type**	**Scan time**	**Histology**	**Results/Remarks**
Helpern et al., 2004	T_2_ with MSSE, T_1_ with inversion prepared, segmented TurboFlash	7T	Tg2576, PS1, PS/APP and WT mice (9x each)	*In vivo*	?	Not done	Reduced T_2_ in HC, cingulate and retrosplenial cortex of PS/APP c.f. WT, no changes in T_1_ and proton density
				*Ex vivo*			
Falangola et al., 2005a	T_2_ multi-slice single SE	7T	PS/APP mice, PS mice and WT	*In vivo*		Results published separately Falangola et al., 2005b	Reduction of T_2_ seen on cortex of PS/APP mouse, statistically small differences between PS and WT mice
Vanhoutte et al., 2005	3D T^*^_2_-weighted GRE imaging, T_2_* mapping	7T	4xAPP^Lon^ mice, 4xWT mice	*In vivo*	?	Thioflavin S for amyloid, Prussian blue for Fe	Reduction in T^*^_2_ values in ventral thalamic nuclei, which contain hypointensities; visual co-registration of histology and imaging
				*Ex viv*o			
Braakman et al., 2006	T_2_-weighted RARE; MSME for T_2_ mapping and changes	9.4T	5xTg2576 mice at 12 and 18 months	*In vivo*	25 min for T_2_ RARE	Immunohisto-chemical staining for plaques, DAB-enhanced Prussion blue for Fe	T_2_ time decreased with age, good correlation between MRI and immunohistology
				Longitudinal			
Borthakur et al., 2006	2D GE T_1_ρ for plaque imaging, T_1ρ_ relaxometric maps	4.7T	2xAPP/PS1, 2xNTg mouse	*In vivo*	3 h	Thioflavin S and immunohisto-chemical staining for plaques	Significant decrease in T_1ρ_ in cortex and HC at 12- and 18-months c.f. controls
El Tannir El Tayara et al., 2006	T_2_ measurements with MSME, T_1_ with 11 sets of IR, parametric T_1_ and T_2_ maps	4.7T	APP/PS1 mice (10x “adults” and 13x “old”) and PS1 mice (9x “adults” and 13x “old”) (control)	*In vivo*	MSME—8 min 49 s, IR 1 h 57 min	Congo Red for amyloid, Perls-DAB for Fe	T_1_ negatively correlated with age, T_2_ in subiculum lower than control, linear relationship between mean Fe load and amyloid load in subiculum of APP/PS1
El Tayara Nel et al., 2007	T_2_ measurements with MSME for parametric T_2_ maps, IR for localizing ROIs	4.7T	11x young APP/PS1 (9–14 weeks age) with no iron on histology	*In vivo*	8 min 49 s for MSME	Not done	Shorter T_2_ in subiculum APP/PS1 mice with higher amyloid load APP/PS1 mice with lower amyloid load PS1 mice; T_2_ reduced in areas of high amyloid without detectable Fe, suggests other mechanisms cause this effect
Falangola et al., 2007	T_2_ measurements with multi-slice single SE, ROIs manually drawn around HC and cortex	7T	Tg2576, PS1, PS/APP and WT mice	*In vivo*	–	Not done	Significant age-related T_2_ reduction in all three Tg mice but not WT, T_2_ reductions in AD models with different extents of amyloid pathology suggests diverse biological mechanisms, likely both Aβ-dependent and Aβ-independent
				Longitudinal			
Thiessen et al., 2010	High-resolution T_2_-weighted MSSE, DWI (MP Turbo-FLASH)	7T	7xTgCRND8 mouse (double human mutant APP—Swe + Ind) + 4 WT mice	*In vivo*	T_2_-weighted—1 h 45 min; DWI—1 h 38 min	Congo Red for amyloid	No differences in T_2_, no significant differences on DWI analysis of ADC values between groups, plaque deposition did not coincide with changes in ADC values
Teipel et al., 2011	T_2_-weighted turbo SE, VBA of entire mouse brain	7T	APP/PS1 *in vivo*	*In vivo*	–	Immunohisto-chemical staining for Aβ, DAB-enhanced Prussian blue for iron	VBA showed reduction in T_2_ in deeper cortical layers, HC and CPu of Tg mice, also thalamus, septal nuclei and cerebellum, no significant change in gray matter; reduced T_2_ relaxation time associated with iron accumulated in plaques

ADC, apparent diffusion coefficient; CPu, caudate putamen; DAB, diaminobenzidine; DWI, diffusion weighted imaging; GRE, gradient echo; HC, hippocampus; IR, inversion recovery; MSME, multislice multi-echo; MSSE, multislice spin echo; RARE, rapid acquisition with relaxation enhancement; ROIs, regions of interest; SE, spin echo; VBA, voxel-based analysis; WT, wild type.

El Tannir El Tayara et al. (2006) included histological analysis of amyloid and iron along with measured T_1_, T_2_ and proton density values in a longitudinal study. They found that T_2_ changes correlated with increasing amyloid load. However, T_2_ was also correlated with age and the lack of a significant difference between T_2_ in APP/PS1 mice and PS1 mice in most brain structures suggests other age-related effects that are distinct from amyloid deposition as main factors in T_2_ decrease. Furthermore, mean Fe did not correlate with T_2_ or age, so that Fe may not participate significantly in age-related T_2_ decrease. In another experiment T_2_ was measured in young APP/PS1 mice whose brains exhibited amyloidosis in the subiculum but had no detectable iron accumulation on histology (El Tayara Nel et al., 2007). The reduced T_2_ in this region is seen by the authors to support the role of other mechanisms in this effect, such as the hydrophobicity of dense amyloid aggregates or other tissue alterations. A further longitudinal analysis of quantitative parametric maps of T_2_ by Falangola et al. (2007), in which mice of various AD genotypes (Tg2576, PS1, and PS/APP) were imaged suggested the reduction in T_2_ involves both Aβ-dependent and Aβ-independent mechanisms. In addition, it was suggested that iron could be a more significant factor for reducing T_2_ in older animals from 12 months.

Both Braakman et al. (2006) and more recently Teipel et al. (2011) in their discussions support a role for plaque-associated Fe in reducing T_2_ relaxation times. As with El Tannir El Tayara et al. (2006) they registered T_2_ maps with histological investigations of amyloid and Fe, with Teipel et al. making additional use of automated voxel-based analysis in studying T_2_ differences. The consistent co-localization of Fe with plaques supports the association of Fe accumulation in plaques with reduced T_2_ (Teipel et al., 2011). Vanhoutte et al. (2005) in their study mapped T^*^_2_ relaxation with 3D gradient echo (GRE) imaging and found a reduction in T^*^_2_ values in the thalamus of singly transgenic mice, where the majority of plaques were associated with Fe.

Taking a different approach Borthakur et al. (2006) analyzed the transverse relaxation in the rotating frame, T_1ρ_ and found this was significantly reduced in the cortex and hippocampus of APP/PS1 mice compared to non-Tg mice. Delineating plaques on imaging and histology, they attributed the effect to amyloid burden but noted it may also result from decreased blood flow and blood volume. Another approach was taken by Thiessen et al. (2010), who used DWI to map ADC values in the neocortex and hippocampus of mice encoding two human APP mutations and registering these with histology. They found no significant change in ADC values compared to controls, suggesting Aβ deposition does not contribute to any diffusional changes if and when they are seen.

Zhang et al. (2004) were first to report on identification of amyloid plaques on MRI imaging in AD transgenic mice without exogenous contrast-enhancement (refer to Table 2). Using a three-dimensional T_2_-weighted multiple spin echo (SE) protocol to image *ex vivo* brains, they visualized numerous hypointensities, which corresponded to amyloid deposits seen on histology. At the same time another group were also able to demonstrate plaques on *ex vivo* images using a T_2_-weighted multi-slice fast SE sequence (Helpern et al., 2004; Lee et al., 2004). Advancing on these achievements, Jack et al. (2004) established the feasibility of *in vivo* plaque imaging, acquiring scans of APP/PS1 mice with T_2_-weighted SE and T^*^_2_-weighted GRE. In subsequent longitudinal study they further validated the use of T_2_-weighted SE for imaging amyloid plaques *in vivo*, visualizing plaques in mice at the earliest age of 9 months with a minimum size of 35 μm (Jack et al., 2005). In their comparison of T_2_-weighted SE to T^*^_2_-weighted GRE imaging, they found the disadvantage of longer acquisition time with SE is offset by its superior ability to resolve smaller plaques. Meanwhile the time efficiency of T^*^_2_-weighted GRE is countered by susceptibility artifacts and its overestimation of the size of some plaques (Jack et al., 2004). Nonetheless there have since been several more studies investigating amyloid imaging with GRE sequences as well as further applications of SE protocols (Vanhoutte et al., 2005; Borthakur et al., 2006; Braakman et al., 2006; Faber et al., 2007; Chamberlain et al., 2009; Dhenain et al., 2009; Meadowcroft et al., 2009; Wengenack et al., 2011).

**Table 2:** **Summary of MR imaging studies of amyloid plaques in transgenic AD mouse models**.

**Study**	**Protocol/s**	**Field**	**Model/s**	**Study type**	**Scan time**	**Histology**	**Results/Remarks**
Zhang et al., 2004	3D T_2_ multi-SE with double-echo acquisition	9.4T	2xAPP/PS1, 1xAPP, 2xWT mice	*Ex vivo*	14 h	Congo Red for amyloid plaques	Numerous plaques in frontal cortex, EC and HC; plaque load highest APP/PS1 followed by APP, many plaques too small to see on MR
Lee et al., 2004	T_2_ imaging with multislice FSE	7T	4xPS/APP, 1xPS, 2xNTg mice	*Ex vivo*	2 h	Immunohistochemical staining for plaques; DAB- enhanced Prussian blue for Fe	Plaques in cortex and HC in PS/APP mice, not seen in PS and NTg mice
Helpern et al., 2004	*ex vivo* of PS/APP and PS1 with fast T_2_SE	7T	Tg2576, PS1, PS/APP and WT mice (9x each)	*In vivo*	?	Not done	Plaques detected in cortex on *ex vivo* imaging (*in vivo* imaging done for T_2_ parametric mapping)
				*Ex vivo*			
Jack et al., 2004	T_2_ SE and T_2_ GE	9.4T	APP/PS1 mice	*In vivo*	1 h 7 min for T_2_ SE and 1 h 27 min for T_2_* GE	Thioflavin S for amyloid, Prussian blue for Fe	SE has superior resolution, accurately reflects plaque size, T_2_* GRE reflects plaque iron content, overestimates plaque size
				*Ex vivo*			
Jack et al., 2005	T_2_ SE	9.4T	APP/PS1 and WT mice	*In vivo*	1 h 40 min	Thioflavin S for amyloid, Prussian blue for Fe	20 μm plaques seen at 3 months *ex vivo*, 35 μm plaques at 9 months *in vivo*; plaques staining for amyloid typically stained for Fe; not all plaques seen on MRI seen due to size
				*Ex vivo*			
				Longitudinal			
Vanhoutte et al., 2005	3D T_2_*-weighted GRE imaging, T_2_* mapping	7T	4xAPP^Lon^ mice, 4xWT mice	*In vivo*	?	Thioflavin S for amyloid, Prussian blue for Fe	Plaques in thalamus, occasionally in subiculum; majority of plaques in thalamus positive for iron; only Fe-associated plaques seen
				*Ex vivo*			
Braakman et al., 2006	T_2_-weighted RARE; MSME for T_2_ mapping and changes	9.4T	5xTg2576 mice at 12 and 18 months	*In vivo*	25 min for T_2_ RARE	Immunohistochemical staining for plaques, DAB-enhanced Prussion blue for Fe	Plaque area, number and size increased with time, Fe associated with many but not all plaques
				Longitudinal			
Borthakur et al., 2006	2D GE T_1_ρ for plaque imaging, T_1ρ_ relaxometric maps	4.7T	2xAPP/PS1, 2xNTg mouse	*In vivo*	3 h	Thioflavin S and immunohistochemical staining for plaques	Able to visualize plaques in HC and cortex but not all (some too small
Jack et al., 2007	T_2_ SE	9.4T	APP/PS1 (treated with anti-Aβ antibodies)	Longitudinal		Thioflavin S for amyloid	Feasibility study for longitudinal imaging of APP/PS1 mice treated with immunotherapy, many small plaques not seen, study did not reach statistical significance (short treatment time and few animals)
Faber et al., 2007	*In vivo* low-res 3D GE, *ex vivo* low- and high-res 3D GE, high-res 3D SE, 2D with SE, GE or CRAZED	17.6T	3xAPP(Lon)/ADAM10-dn mice	*In vivo*	*In vivo*—low-res 3D GE 34 min, *ex vivo*—low-res 3D GE 82 s, high-res 3D GE 5 h 50 min, high-res 3D SE 8 h 36 min	Immunohistochemical staining for amyloid, Prussian blue for Fe	Large iron-containing plaques seen in thalamus *in vivo* and *ex vivo* with 3D GE, cortical plaques not seen (lower iron content); contrast added with CRAZED (technique prone to false positives CRAZED, unsuitable for routine us
				*Ex vivo*			
Chamberlain et al., 2009	T_2_, T_2_* and SWI; sequences used: SE, FSE, mSE, maSE, GE, mGE, mASE with SWI, GE with SWI, mGE with SWI	9.4T	4xAPP/PS1 mice (9 month old), 1xAPP mouse (22 month old) and 2xWT mice	*Ex vivo*	Long	Thioflavin S for amyloid and DAB for Fe	Multi-echo gives better CNR than single-echo, SWI increased CNR; summed echo better than FSE (blurring), T_2_* better CNR than T_2_ (SWI provides greatest CNR), not predictable on histology which plaques will have increased CNR; *in vivo* GE and SWI in mice impractical (susceptibility artifacts), could be feasible in humans (increased distance from air/tissue interface)
Dhenain et al., 2009	T_2_* GE (all mice), T_1_ 3D GE (some), T_2_ 3D SE (most *in vivo* mice); T_2_* GE for all brains	4.7T	APP/PS1, PS1 and C57BL/6 (WT) mice	*In vivo*	51 min for *in vivo*, 7–8 h for *ex vivo*	Congo Red for plaques, DAB-enhanced Perl's for Fe, TEM analysis, elemental analysis with SIMS	Thalamic plaques, related to high iron and calcium load
				*Ex vivo*			
Meadowcroft et al., 2009	T_2_* multi-GE using histological coil	7T	5xAPP/PS1, 3xNTg control mice	*Ex vivo*	6 h 32 min	Co-stained for both iron and amyloid with DAB-enhanced Perl's Prussian Blue stain followed by Thioflavin S, TEM on samples of regions with Aβ plaque distribution	T_2_* contrast in humans from higher iron deposition in plaques; little iron in APP/PS1 mouse plaques, denser structure excluding Prussian blue reaction
Wengenack et al., 2011	T_2_-weighted SE, 3D T_2_-weighted GE	9.4T	APP/PS1 mice	*Ex vivo*	SE 1 h 42 min, GE 1 h 22 min	DAB-enhanced Prussian blue for Fe, Thioflavin S and immunohistochemical staining for Aβ	Many plaques in cortex, HC and thalamus, increasing number and size with age; cortical and HC plaques develop at 3 months, thalamic plaques at 12 months, less Fe in cortical and HC plaques, overall Fe content in APP/PS1 higher than in WT; T_2_SE more reliable for cortical and HC plaques

CNR, contrast to noise; CRAZED, COZY revamped with asymmetric z-GE detection; DAB, diaminobenzidine; EC, entorhinal cortex; FSE, fast spin echo; GE, gradient echo; HC, hippocampus; mGE, multiple gradient echo; maSE, multiple asymmetric spin echo; mSE, multiple spin echo; MSME, multislice multi-echo; MSSE, multislice spin echo; RARE, rapid acquisition with relaxation enhancement; SE, spin echo; SIMS, secondary ion mass spectroscopy; SWI, susceptibility weighted imaging; TEM, transmission electron microscopy; WT, wild type.

One of the main aims of the MR studies is to reduce the scanning time required. Zhang et al. (2004) required 14 h to acquire their images while Lee et al. (2004) managed to scan *ex vivo* mouse brains in 2 h, a time more compatible with *in vivo* mouse imaging. Jack et al. acquired *in vivo* T_2_-weighted SE and T^*^_2_-weighted GRE in 1 h 7 min and 1 h 27 min respectively. The time efficiency of GRE sequences has meant *in vivo* scanning times of less than an hour have been achieved (Faber et al., 2007; Dhenain et al., 2009). Faber and co-workers, for example, were able to achieve significant gains in time efficiency with the use of bright contrast obtained by correlated spectroscopy (COZY) revamped with asymmetric z-GRE detection (CRAZED) sequences. However, in these studies only thalamic plaques could be visualized and few if any cortical plaques were seen (Faber et al., 2007; Dhenain et al., 2009). On the other hand, Braakman et al. (2006) imaged cortical plaques using a T_2_-weighted rapid acquisition with relaxation enhancement (RARE) sequence that resulted in a scan time of 25 min.

Thalamic plaques are more readily identified on MR imaging (Dhenain et al., 2009). This has been attributed to variations in tissue Fe deposition and Fe density in plaques (Jack et al., 2004; Faber et al., 2007; Dhenain et al., 2009). This leads to the question of the precise mechanisms by which transverse relaxation is changed leading to contrast in plaques relative to surrounding brain tissue on imaging. Comparing DAB-enhanced Perl's staining for Fe with the distribution of amyloid deposits in histology of mice brains shows they are frequently co-localized, indicating Fe association with plaques (Jack et al., 2004; Lee et al., 2004; Vanhoutte et al., 2005; Braakman et al., 2006). Thalamic plaques have high concentrations of Fe associated with them (Faber et al., 2007; Dhenain et al., 2009). Higher Fe concentrations produce a “blooming effect” on T^*^_2_-weighted imaging, which can lead to overestimation of plaque size (Jack et al., 2004). However, plaques in the cortex and hippocampus of AD mice have less associated Fe than thalamic plaques in mice and amyloid plaques in human AD brains (Vanhoutte et al., 2005; Faber et al., 2007; Leskovjan et al., 2009, 2011; Meadowcroft et al., 2009; Wengenack et al., 2011). Illustrating this difference, in the studies using T^*^_2_-weighted GRE sequences only thalamic plaques were clearly visualized (Vanhoutte et al., 2005; Faber et al., 2007; Dhenain et al., 2009). The difference in size, morphology, mineral content and amyloid fibrillar structure in these thalamic plaques and their lack of correlation with brain amyloidosis make them of little use in the development of MR imaging (Dhenain et al., 2009; Chamberlain et al., 2011; Wengenack et al., 2011). Nonetheless the influence of Fe susceptibility effects on T_2_ relaxation is still regarded as a major factor in achieving contrast when visualizing plaques in the cortex and hippocampus of AD transgenic mice (Helpern et al., 2004; Jack et al., 2004; Lee et al., 2004; Zhang et al., 2004; Braakman et al., 2006; Chamberlain et al., 2009; Meadowcroft et al., 2009; Wengenack et al., 2011).

Conversely, the fact that Fe is much reduced in amyloid plaques in mice and secondly that not all visualized plaques contain Fe suggest that there may be other sources of MR contrast in amyloid plaques. Accelerated T_2_ relaxation in plaques may be partially due to exchange between tissue water protons and protons in plaque-associated proteins (Jack et al., 2004) or impaired cell physiology with resultant reduced cerebral blood flow (Helpern et al., 2004; Borthakur et al., 2006). It has also been suggested that reduced T2 signal may be due to exclusion of water molecules from the dense hydrophobic core of amyloid deposits (Lee et al., 2004; Wengenack et al., 2011), though the fact that the proton density in plaques is only slightly reduced compared to normal cortical tissue does not lend this support to this proposition (Chamberlain et al., 2009; Wengenack et al., 2011). Part of the difficulty in understanding the properties of plaques in MR imaging has been due to the imprecise nature of co-registering the plaques seen on imaging with histological localization of amyloid deposits and Fe. Meadowcroft et al. overcame this difficulty by using a coil optimized for scanning histological sections, allowing very accurate co-registration between images and histology. Thus, they were able to directly compare the difference in Fe content in plaques in APP/PS1 mice with human AD brain tissue and control mice and analyse its effect on T_2_*-weighted imaging. Iron was shown to play a role in the contrast in amyloid in human AD brains on imaging, yet plaques in APP/PS1 mice were as easily observed despite having significantly less Fe. This plaque-associated signal loss may be explained by the interactions of water with the highly compacted amyloid fibril mass (Meadowcroft et al., 2009).

Attempts to enhance the endogenous contrast in plaques with improved scanning efficiency and reliability has resulted in a wide range of image acquisition techniques being investigated. These have involved T_2_-weighted SE sequences and T^*^_2_-weighted and T_1ρ_ GRE sequences, and included methods for contrast enhancement such as susceptibility-weighted imaging (SWI) (refer to Table 2). In a comparison of multiple techniques applied to mouse brain imaging by Chamberlain et al. (2009), T^*^_2_ was shown to give superior plaque contrast-to-noise ratio (CNR) compared to T_2_ while GRE gave consistently higher CNR compared to SE. They further investigated the combination of SWI with multiple asymmetric SE, GRE and multiple GRE to increase plaque contrast. As with CRAZED, SWI relies on Fe for contrast enhancement. They found that SW1 sequences provided the greatest CNR on *ex vivo* imaging of cortical plaques. However, the use of GRE and SWI *in vivo* is complicated by susceptibility artifacts caused by the air-to-tissue and/or fat-to-skull interfaces. Jack and co-workers consistently found T_2_-weighted SE sequences more reliable in the detection of plaques as they are less reliant on their Fe content (Jack et al., 2004; Chamberlain et al., 2009; Wengenack et al., 2011).

One of the main concerns with direct imaging of amyloid plaques is the lack of specificity in the signal changes that might correspond to Aβ deposits (Poduslo et al., 2002; Jack et al., 2004). T_1_ and proton density values do not differ significantly between amyloid deposits and normal brain tissue (Helpern et al., 2004; Chamberlain et al., 2009). On the other hand Fe, a major source of accelerated T_2_ and T^*^_2_ decay is non-specific. Consequently blood vessels or microhaemorrhages can be evident in T2^*^ and SW1 images and thus also seen in normal brains (Poduslo et al., 2002; Jack et al., 2004). A strategy aimed at overcoming these initiatives, and analogous to radionuclide imaging, is the use of amyloid-targeting ligands labeled with MR contrast-enhancing molecules.

One molecule shown to bind with high affinity and specificity to amyloid deposits was Aβ itself (Poduslo et al., 2002; Wadghiri et al., 2003; Sigurdsson et al., 2008; Braakman et al., 2009; Yang et al., 2011). Following Aβ labeled radionuclide experiments, Poduslo and co-workers surmized that labeling with gadolinium (Gd) could enhance MR image contrast by acceleration of T_1_ relaxation and to a lesser extent T_2_ relaxation of nearby water molecules. They were able to demonstrate contrast enhancement of amyloid plaques on both T_1_-weighted and T_2_-weighted images of *ex vivo* mouse brains following injection of synthetic Aβ_40_ chelated to Gd prior to euthanization of the animal model. Following this study Wadghiri et al. investigated the *in vivo* application of enhancement with Aβ_40_ attached to Gd and monocrystalline iron oxide nanoparticles (MION). They were able to detect plaques on *in vivo* T_2_-weighted SE and T^*^_2_-weighted GRE images but T_1_-weighted imaging required prohibitively long scan times. Sigurdsson et al. showed again that Gd is able to enhance contrast on T^*^_2_-weighted imaging while Yang et al. investigated the use of ultrasmall superparamagnetic iron oxide (USPIO) nanoparticles coupled to Aβ_42_ with T^*^_2_-weighted GRE. T_1_-weighted imaging is less sensitive to the magnetic susceptibility effects of Fe and so demonstrated fewer artifacts from Fe-associated structures such as white matter and blood vessels. Consequently there is ongoing research into developing T_1_-weighted sequences for use with Gd-labeled contrast enhancing agents (Wengenack et al., 2008).

Two disadvantages are evident with the use of intravenously administered Aβ peptides to label plaques. Firstly, the impermeable nature of the normal blood-brain-barrier necessitates co-administration of mannitol or complexion of ligands with polyamines such as putrescine to facilitate passage of the ligand into the brain to reach its target (Wadghiri et al., 2003; Delatour et al., 2006; Braakman et al., 2009). Secondly, Aβ is potentially toxic. Sigurdsson et al. (2008) developed a ligand using the truncated Aβ_1−30_ peptide chelated to Gd. Other groups have developed ligands based upon the amyloidophilic compound Congo Red often used in histological staining (Higuchi et al., 2005; Li et al., 2010). In a particularly novel approach, Higuchi et al. (2005) utilized a Congo Red-based compound labeled with ^19^F. This compound was able to cross the blood-brain-barrier and bind specifically to amyloid. ^19^F is MR-sensitive but present in only very low concentrations in biological tissues, resulting in very little non-specific background signal (Higuchi et al., 2005; Braakman et al., 2009). Another strategy to overcome the toxic effects of Aβ ligands is to use anti-Aβ monoclonal antibodies linked to Gd (Wengenack et al., 2008).

In summarizing, the availability of Tg mouse models of AD has greatly accelerated the progress made in MR amyloid imaging by providing a convenient and efficient platform for developing techniques. In particular, the ability to correlate acquired MR images with histology has been invaluable to understanding pathophysiology and its MR behavior. Despite the limitations so far, the advances that have been made show a greater potential that remains achievable as technology and techniques improve.

### Positron emission tomography imaging

Following the development of functional FDG PET imaging in patients with AD, the feasibility of detecting and monitoring similar changes in cerebral metabolism in AD mouse models was investigated (Reiman and Caselli, 1999; Valla et al., 2002, 2006, 2008; Dubois et al., 2010; Nicholson et al., 2010). FDG autoradiography in the brains of a variety of AD mouse models, ranging from single transgenic to triple transgenic mice, revealed hypometabolism in the posterior cingulate cortex consistent with findings in human studies (Reiman and Caselli, 1999; Valla et al., 2002, 2006, 2008; Nicholson et al., 2010). However, these findings did not translate to any detectable change on *in vivo* FDG PET in mice (Kuntner et al., 2009).

Furthermore, amyloid imaging in mouse models of AD has not shown the same effective results as has been achieved in humans. This contrasts with MRI studies, where conversely promising findings in animal studies have yet to translate to consistent human application. Initial PET studies using PIB in transgenic AD mice did not detect any difference in ligand retention between control mice and either Tg2576 or APP/PS1 (Klunk et al., 2005; Toyama et al., 2005). The lack of significant difference has been explained by reduced cerebral perfusion along with a reduction in the density of high affinity ligand binding sites in the plaques in transgenic mice (Klunk et al., 2005; Toyama et al., 2005; Kuntner et al., 2009; Higuchi et al., 2010). The latter may be attributed to the co-deposition of both rodent and human Aβ fibrils in mouse plaques, disrupting the formation of high affinity binding sites (Ye et al., 2006; Higuchi et al., 2010). Similarly, imaging with the probe FDNNP in Tg2576 mice did not differentiate them from control mice (Kuntner et al., 2009), despite its successful application in a triple transgenic rat model of AD (Kepe et al., 2006; Teng et al., 2011).

One way of overcoming the reduced density of high affinity binding sites is by the administration of PIB synthesized with high specific radioactivity, thereby reducing blockage of sites by non-radioactive ligands and improving the plaque specific signal (Maeda et al., 2007; Higuchi, 2009). This method has enabled demonstration of amyloidosis with PIB in the hippocampus and neocortex of transgenic APP23 mice compared to age-matched controls (Maeda et al., 2007, 2011; Higuchi, 2009). In turn, the ability to image amyloid *in vivo* in transgenic mice enables longitudinal studies for monitoring the effectiveness of anti-amyloid interventions (Maeda et al., 2007, 2011; Higuchi, 2009). Also, combining amyloid imaging with other PET ligands, such as those specific for markers of neuroinflammation, can help further the understanding of the associated molecular and cellular mechanisms of AD pathology and experimental therapeutic approaches (Maeda et al., 2007, 2011; Higuchi, 2009; Higuchi et al., 2010). Studies exemplifying this have demonstrated a decline in hippocampal amyloid following passive immunization with anti-Aβ antibodies in transgenic mice, while concurrently revealing associated microglial activation and increased neuroinflammation (Maeda et al., 2007, 2011; Higuchi, 2009).

Notwithstanding these important advances, the use of PET in preclinical small animal imaging in AD remains constrained by technical considerations. The spatial resolution, of current generation scanners, which can range from 0.5 to 3 mm, is insufficient in the context of the small size of mouse brain structures (Nikolaus et al., 2004; Kuntner et al., 2009; Dubois et al., 2010). Difficulties also arise from partial volume error that affects volume definition and radioactivity measurements in small volumes of interest (Nikolaus et al., 2004; Kuntner et al., 2009).

### Computed tomography imaging and magnetic resonance spectroscopy

The superior resolution of MR for visualizing brain tissue has meant CT has been seldom utilized as an option for imaging amyloid pathology in mice. Nonetheless, a few studies have explored CT neuroimaging. Hyde et al. (2009) combined structural information from CT with fluorescence molecular tomography with a fluorescent oxazine dye for *in vivo* quantification of amyloid plaque burden in APP23 mice. Others have applied CT directly to imaging amyloid plaques, either with phase-contrast X-ray CT (Noda-Saita et al., 2006) or with diffraction-enhanced imaging (DEI) in micro-CT mode (Connor et al., 2009). Both Noda-Saita et al. (2006) and Connor et al. (2009) demonstrated individual plaques on *ex vivo* imaging with high spatial resolution comparable to histological analysis. However, the high level of irradiation associated with phase-contrast CT and its inability to image the brain within the skull meant it could not be applied *in vivo* (Noda-Saita et al., 2006). According to Connor et al. DEI could overcome the issue of *in vivo* imaging by using higher energy x-rays to reduce attenuation from the skull. Nonetheless, these limitations of plaque imaging with CT and the lack of any clear advantage over MR plaque imaging has resulted in no studies being published to date with *in vivo* CT imaging in AD mouse models.

In contrast, MRS has had greater utility in evaluating pathological changes in a number of AD mouse models (Marjanska et al., 2005; von Kienlin et al., 2005; Oberg et al., 2008; Chen et al., 2009; Salek et al., 2010; Xu et al., 2010). Analysis of the metabolic profile of PS2APP (von Kienlin et al., 2005) and APP/PS1 mice (Marjanska et al., 2005; Oberg et al., 2008) by *in vivo* MRS revealed significant decreases in the NAA and glutamate levels relative to Cr with advancing age. These changes in transgenic models, in particular the APP/PS1 model are concordant with those seen in human AD brains (Marjanska et al., 2005; Michaelis et al., 2009). Marjanska and co-workers also observed an increase with age in the concentration of myo-inositol (m-Ins), a marker of glial activation, in APP/PS1 mice compared to wild-type controls. This finding was later confirmed by Chen et al. (2009) and has confirmed the significance of m-Ins as an alternative metabolic marker to NAA, enabling investigation of other disease processes in AD (Oberg et al., 2008). Taken together the results from these studies provide good support for the role of MRS in preclinical research in AD.

## Current status of neuroimaging biomarkers in Alzheimer's disease

Technological advances in neuroimaging have yielded a burgeoning wealth of information on the pathology of AD. Coupled with our growing understanding of the molecular and cellular basis of the disease, these advances have opened new possibilities and potential for new approaches. Foremost of these advances has been that of PET, which has exemplified the versatility of neuroimaging in both preclinical and clinical research in AD. Early development of ligands specific for neuroreceptors revealing changes in neurotransmitter systems, such as the cholinergic system and dopaminergic system, together with functional imaging enabled by FDG-PET, provided initial insights into the molecular mechanisms of AD (Vallabhajosula, 2011). However, the development of amyloid-binding probes has markedly enhanced the potential for PET imaging in the diagnosis of AD. The initial ligand synthesized for this purpose, FDNNP, which exhibits non-specific binding to both amyloid and NFT pathology, was quickly surpassed by the amyloid-specific ligand [^11^C]-PIB. Since the introduction of PIB, *in vivo* amyloid imaging has demonstrated the capacity to diagnose AD with good accuracy and to differentiate AD from dementia due to a number of other causes. It has also provided immense insights into clinicopathology, revealing key information that has greatly influenced our current understanding of the amyloid hypothesis of AD pathogenesis. The lack of correlation between amyloid burden and clinical severity in AD, along with the significant proportion of cognitively normal elderly with detectable amyloid, has recently resulted in the presence of cerebral amyloid being viewed in an increasingly complex manner in disease development. That Aβ pathology plays a key role remains widely accepted, but the pathological mechanisms of AD may occur prior to the formation of amyloid plaques. Nonetheless, the utility of amyloid imaging is firmly established and is reflected by ongoing development into amyloid-targeting ligands (Vallabhajosula, 2011), particularly ^18^F-labeled ligands that exhibit more specific binding and have greater clinical availability due to the 2 h half-life.

The evolution of MRI techniques has also provided important new perspectives for the development of MRI biomarkers in AD. There are now a number of quantitative structural MRI studies demonstrating volume loss in the medial temporal lobe, particularly the hippocampus, as a significant and early marker of disease development in AD (Ewers et al., 2012) [a recent study using MR imaging in infants (2–25 months of age) of different apolipoprotein E status revealed that APOE E4 carriers had decreased gray matter volume in areas typically affected in AD, raising the possibility that a genetic predisposition to AD is reflected by early anatomical alterations in the brain (Dean et al., 2014)]. The neuronal loss underpinning the macroscopic regional gray matter atrophy seen on T_1_-weighted MRI can be further investigated at the microscopic level, by techniques such as MTR and MRS. In a recent study of quantitative parametric mapping of MTR parameters and gray matter volumetric maps in AD patients, Giulietti et al. (2012) suggest that the reduction in MTR may reflect not only amyloid deposition, but also metabolic derangement due to mitochondrial dysfunction, as the affected regions exhibit both PIB accumulation and reduced glucose metabolism on FDG-PET. Hence MTR can give information complementary to conventional structural MRI, which may have further value in investigating other groups such as MCI subjects and other dementia patients. Similarly MRS, by enabling detection of metabolites reflecting neuronal loss and gliosis can provide further information on pathophysiological changes to complement structural imaging changes in MCI and early AD. Using this multimodal approach, Westman et al. (2011) in their multivariate analysis of MRI volumetric measures and MRS measures improved the discrimination between early AD patients and healthy controls. DTI has also been used to reveal damage to white matter tracts in and between known affected areas in AD, such as in the posterior cingulum and between the prefrontal cortex and the medial temporal lobe or parietal cortex. Advanced MRI techniques have been applied to reveal important information on the consequences of neuronal dysfunction and neuronal loss. Axonal degeneration and white matter changes that occur secondary to neuronal loss can be investigated with DTI. Yukashev and colleagues demonstrated that both macroscopic changes shown by volumetric analysis and microstructural changes revealed by DTI lead to metabolic changes in the posterior cingulate cortex (Yakushev et al., 2011a,b). Hattori et al. (2012) utilized DTI to analyse the fornix, which contains efferent fibers from the hippocampus, in AD patients and patients with normal pressure hydrocephalus (NPH) which is another cause of progressive dementia. Their tract-specific analysis revealed differing patterns of damage between NHP and AD patients, suggesting the potential of this approach in differentiating these two causes of dementia. Functional MRI, measuring changes in regional brain activity arising from gray and white matter changes and particularly loss of functional connectivity, is another emerging MRI technique able to complement structural MRI. In their analysis of volumetric gray matter change and resting state fMRI measurements, Dai et al. (2012) were able to discriminate between early AD patients and healthy controls with an accuracy of 89.5%. However, validation of these MRI techniques, applied individually and in multimodal approaches requires further investigation in larger groups (Drago et al., 2011).

**Figure 1:**
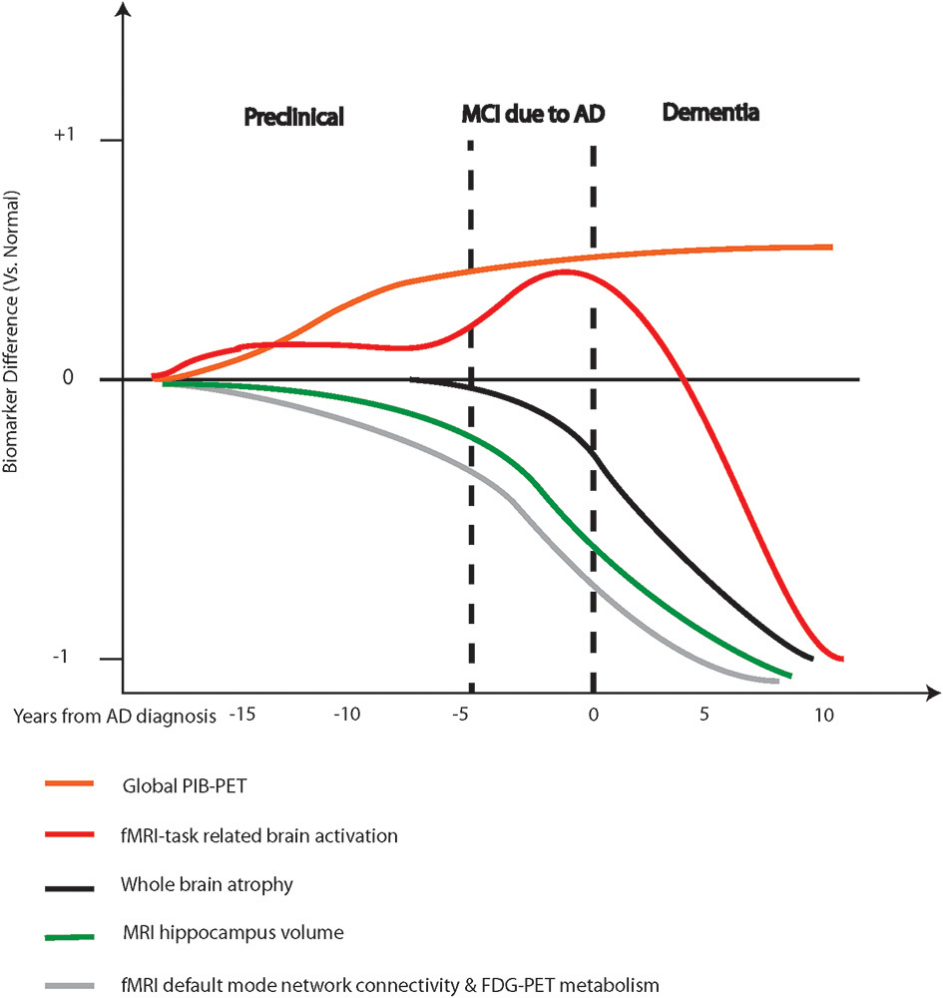
**Hypothetical model of various neuroimaging biomarkers and their predicted utility during disease progression**. Adapted from Ewers et al. (2011) and Frisoni et al. (2010).

Recent research publications have shown a clear evolution from single-modality imaging to multimodality imaging studies of neuroimaging biomarkers of AD. The complementary information yielded by multiple imaging approaches gives greater insight into the pathophysiological processes occurring *in vivo*. An improved understanding of the temporal relationship of emerging neuroimaging biomarkers is also important. The dynamic model of imaging biomarkers in AD (Frisoni et al., 2010; Jack et al., 2010; Cavedo and Frisoni, 2011; Ewers et al., 2012) reflects the developing concept of AD as a continuum of pathophysiological processes, with clinical AD being a late manifestation (refer to Figure 1). Amyloid imaging not only supports the classification of amnestic MCI as the prodromal stage, but also provides evidence for an asymptomatic preclinical stage in which the pathological processes for AD have already been triggered (Cavedo and Frisoni, 2011; Ewers et al., 2012). PIB imaging has revealed up to 50% of MCI patients and up to 30% of cognitively normal elderly have significant amyloid deposition, associated with changes in brain structure and function (Ewers et al., 2012; Mormino et al., 2012). In MCI, both PIB positivity and the presence and rate of hippocampal atrophy on MRI are predictive of conversion to clinical AD. However, as reflected in the dynamic imaging biomarker model, the amyloid burden has reached a plateau in MCI and hippocampal atrophic changes are already significant following the onset of clinical AD (Cavedo and Frisoni, 2011; Ewers et al., 2012). This suggests that biomarkers have less relevance in the latter stages of the disease continuum. Furthermore, PET imaging and structural MRI do not offer any advantage in accuracy and utility over clinical criteria for AD diagnosis, hence part of the reason why they are yet to be accepted as part of diagnostic criteria (McKhann et al., 2011).

Consequently, the focus of neuroimaging has now shifted to defining the preclinical stage of AD (Sperling et al., 2011). As mentioned, a proportion of cognitively normal individuals are known to harbor significant amyloid as evidenced by PIB retention, with the onset of this deposition occurring after the age of 50 in people without familial AD (Ewers et al., 2012). It is possible that slight elevations in PIB in cognitively normal elderly may have biological relevance (Mormino et al., 2012). When studied with FDG-PET, cognitively normal individuals carrying the ApoE ε4 demonstrate early abnormalities (Ewers et al., 2012). Similarly, PIB-positive elderly may demonstrate lack of deactivation and disrupted functional connectivity in the DMN with fMRI, and greater atrophy in the hippocampus and cingulate cortex on structural MRI (Ewers et al., 2012). Consequently, defining preclinical AD has been a major focus, leading to recommendation of a research framework for working toward this goal by the National Institute on Aging—Alzheimer's Association workgroups (Sperling et al., 2011). Neuroimaging is an integral component in developing the preclinical AD definition, as reflected in the recommended draft operational research framework. Further research, particularly longitudinal studies, are needed to contribute toward elucidating the full continuum of AD pathophysiology.

Our understanding of the pathological mechanisms in AD has been advanced by the extensive application of neuroimaging techniques to visualize and understand *in vivo* neuropathology. The role of synaptic failure in AD has informed studies in fMRI, while neuroinflammation and mitochondrial dysfunction is important in interpretation of MRS and MTR findings. Additionally, the role of metal dyshomeostasis in AD pathogenesis has suggested other approaches to imaging. Variations in Fe distribution in the brain are detectable by MRI (Haacke et al., 2005; Bartzokis et al., 2007), thereby suggesting it can be potentially be applied to study the analysis of Fe dysregulation in AD. Techniques to analyse the distribution of Fe through variations in magnetic relaxivity (R and R^*^) values have been carried out in post-mortem AD brains (Antharam et al., 2012). Nakada et al. (2008) utilized SWI in MRI on a 7T scanner to visualize plaque-like pathology in patients with AD. However, their sample size was small and their study could not be correlated with histology. Studies of other aspects of AD pathology, such as the strengthening evidence for the primary role of Aβ oligomers in neurotoxicity and the importance of NFTs in the pathology cascade, will require novel neuroimaging developments. Further insights into the molecular aspects of AD may provide opportunities for clinical imaging applications, and enable further progress on defining preclinical AD. Ultimately, neuroimaging based upon AD patholophysiology may bring about the realization of accurate early diagnosis, thereby providing the opportunity for early preventative interventions, which can be monitored for efficacy by tracking changes in disease processes using neuroimaging techniques.

## Translating neuroimaging: understanding and integrating pathophysiological mechanisms in mouse models

Transgenic AD mouse models are imperfect replicas of the human disease. This has significant implications for the application and interpretation of current neuroimaging findings in animal models. The expression of human Aβ in mice affects the physicochemical properties of the formed plaques, which contain both human and rodent Aβ. Apart from altered fibril structure, mouse plaques differ in the concentration of the associated metals, in particular their Fe content. Furthermore, these models fail to reproduce the full spectrum of AD pathology evident in human brains, suggesting that other factors or key pathological mechanisms are missing. For example, single transgenic mice do not exhibit neuronal loss despite Aβ deposition, though this is changed with the introduction of multiple genes (Elder et al., 2010). Also, NFTs are not found in transgenic AD mice unless a tau mutation is introduced (Elder et al., 2010; Balducci and Forloni, 2011), However, these triple-transgenic mice may not be wholly relevant to AD as tau mutations are not a feature of AD. It may be that the much shorter lifespan of mice precludes key necessary pathological processes associated with human aging, or alternatively these variations may again be attributed to differences between mouse and human proteins (Elder et al., 2010). Another suggestion is, due to their limited AD phenotype, transgenic mice may instead represent models of preclinical AD (Woodhouse et al., 2009; Zahs and Ashe, 2010). In either case, the transgenic mouse model is non-etheless an invaluable asset in the investigation of the pathophysiological mechanisms of the disease. Mouse studies have helped elucidate the role of Aβ accumulation and other pathological processes, including oxidative stress, inflammation and metal dyshomeostasis in the development of neuropathology (reviewed in Balducci and Forloni, 2011). Bidirectional translation between human research and preclinical studies in mice is also fundamental in developing our understanding of AD and furthering advances in neuroimaging biomarkers (Higuchi et al., 2010). Furthermore, the time efficiency and cost-effectiveness afforded by the accelerated formation of neuropathology and their short lifespan, makes them particularly useful for longitudinal neuroimaging studies (Strome and Doudet, 2007; Waerzeggers et al., 2010).

The most extensive use of mouse models for neuroimaging in AD has been in MR imaging studies. The technical difficulties of applying cognitive paradigms to provoke responses in rodents and the need to anaesthetize them for scanning has meant that only a few fMRI studies have been reported (Benveniste et al., 2007), The detection of brain atrophy in transgenic mice has varied between structural MRI studies, and atrophic changes do not appear to correlate with the degree of Aβ deposition (Balducci and Forloni, 2011). Furthermore, the pattern and mechanisms of atrophy in mice appear to differ from those in human AD (Delatour et al., 2006; Maheswaran et al., 2009). These inconsistencies in structural imaging studies may be addressed using newer mouse models, such as the APPxPS1-Ki mouse, which exhibits both early onset amyloid-β deposition and subsequent neuronal loss (Faure et al., 2011). Other studies, using MRS in transgenic mice to investigate changes in the metabolites NAA, choline and mIns, have closely paralleled findings from human studies (Barba et al., 2008), and have provided important insights into the progression of pathological changes. MRS changes are associated with areas exhibiting amyloid deposition (Salek et al., 2010; Xu et al., 2010), though altered metabolites may be detectable in mice before accumulation of plaques and major histological changes, at an early stage of the disease.

**Figure 2:**
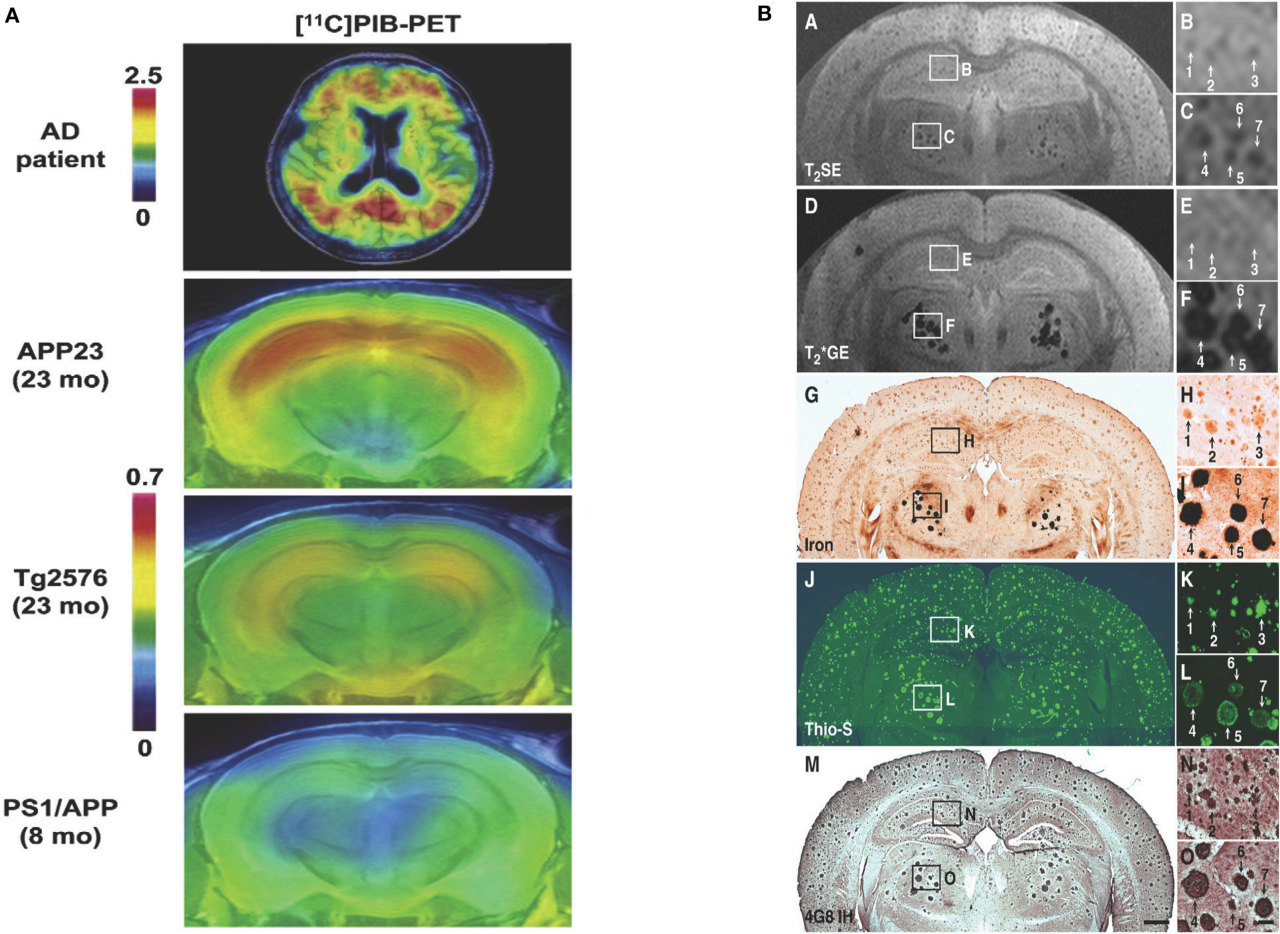
**(A)** [11C]-PIB-PET images illustrating amyloid depositions in brains of an AD patient (axial view) and different strains of APP transgenic or PS-1/APP double transgenic mice (coronal view) merged onto anatomical MRI maps. Human and mouse images are differently scaled according to the maximal binding potential for the radiotracer in each species, as indicated by vertical bars from Higuchi et al. (2010). **(B)** Five-way anatomic spatial coregistration of a 24-month-old APP/PS1 AD transgenic mouse brain. *Ex vivo* MRI scans of matched sections imaged using either a (A) T_2_SE or (D) T^*^_2_ GE pulse sequence. Matched adjacent histological sections processed with (G) DAB-enhanced iron staining, (J) thioflavine S amyloid staining, or (M) anti-Aβ peptide immunohistochemistry. Scale bar = 500 μm. (B,E,H,K,N) Higher magnification of hippocampal plaques positively matched by spatial coregistration. Corresponding plaques are labeled with numbers when present in a particular section. (C,F,I,L,O) Higher magnification of thalamic plaques positively matched by spatial coregistration. (O) Scale bar = 100 μm from Wengenack et al. (2011).

By far the majority of endeavors using MRI in transgenic mice have been for developing imaging of amyloid plaques (see Figure 2B). Various image acquisition protocols have now been investigated to improve the resolution and the plaque-specific contrast and to reduce scanning times. Techniques sensitive to the susceptibility effects of plaques, T_2_- and T^*^_2_-weighted imaging are able to provide good contrast for plaque imaging, while further enhancement with SWI has the potential to shorten the duration of scanning to clinically acceptable times before MR amyloid imaging is translated from preclinical mouse studies to human clinical research. Despite promising results from numerous studies, a number of issues are still to be resolved. The majority of mouse imaging studies utilize ultra-high field strength magnets of 9.4 Tesla, while most clinical scanner magnets are 1.5 or 3 Tesla. Excluding susceptibility artifacts, arising from other structures giving similar signal on T_2_- and T^*^_2_-weighted images or from the skull-air interface on SWI, is a significant technical challenge. Also, the relationship between the MRI signal and the intrinsic biochemical properties of amyloid plaques needs further clarification. Mouse plaques contain less Fe than human plaques (Leskovjan et al., 2009; Meadowcroft et al., 2009), which suggests different mechanisms are responsible for the decay of transverse relaxation in mouse plaques compared to human plaques (Meadowcroft et al., 2009). Alternatively, enhancing contrast with plaque-specific contrast agents could circumvent the reliance on the intrinsic susceptibility signal of plaques using MRI. As with PET ligand development, mouse models lend themselves readily to the development and testing of plaque specific contrast agents.

In contrast to MRI imaging, the PET amyloid imaging has been less effectively translated to transgenic mouse models (see Figure 2A). As previously mentioned, a major reason for this is the paucity in high affinity ligand binding sites in mouse amyloid, which can partly be overcome by synthesizing ligands with higher specific radioactivity (Waerzeggers et al., 2010). Improving the spatial resolution of preclinical PET scanners is another challenge. Most studies of new PET ligands targeting amyloid have relied on *ex vivo* autoradiography to localize ligand binding in the brains of transgenic mice (Ono et al., 2011; Vallabhajosula, 2011). Yousefi et al. (2011), in their evaluation of [^11^C]-Labeled Imidazo[2,1-b]benzothiazoles as PET tracers, demonstrate that improvements in small animal PET scanners have enabled *in vivo* imaging in mouse models to contribute toward ligand development. Furthermore, *in vivo* PET in mice provides immense potential for furthering the understanding of pathological mechanisms and development of ligands specific for AD neuroimaging (Higuchi et al., 2010; Klohs and Rudin, 2011). Multi-tracer studies have been particularly valuable in elucidating pathological relationships in mice, such as the loss of noradrenergic neurons and inflammatory reactions with decreased metabolism (Waerzeggers et al., 2010). Maeda et al. (2007) demonstrated not only the feasibility of PET amyloid imaging of transgenic mice using PIB with high specific radioactivity, but also combined PIB with PET markers for glial activation application in longitudinal monitoring of immunotherapy in mice. Their subsequent study investigating glial responses in mouse models of AD and tau pathologies with PET offered important insights into the pathological mechanisms at work (Maeda et al., 2011). In particular, their findings suggest a link between upregulation of microglial inflammatory responses by Aβ deposits and conversion of glial responses to a neurotoxic form mediating tau pathology and neuronal loss (Higuchi et al., 2010; Maeda et al., 2011). This may explain the absence of neuronal loss seen in AD mouse models that lack tau pathology (Higuchi et al., 2010). The stimulation of specific immune responses by Aβ and its adverse effect on tau pathology and neuronal survival has implications for immunotherapy in the treatment of AD (Higuchi et al., 2010; Maeda et al., 2011). These findings highlight the value of PET imaging in the evaluating therapeutic interventions, by enabling key pathophysiological mechanisms to be monitored.

As neuroimaging in AD mouse models advances in parallel to clinical research, new imaging approaches will be informed by developments in our understanding of AD pathophysiology. Structural MRI has gained new perspectives from incorporating DTI and MTI, by elucidating early microstructural changes that can be correlated with the development of atrophic changes. Analysis methods such as voxel-based morphometry and deformation-based morphometry are being used to study subtle atrophy in vulnerable brain regions in AD transgenic mice. MR amyloid imaging can be expected to further advance, with ongoing developments in amyloid specific MR contrast agents. For example, a novel recent approach taken by Higuchi et al. (2005) and further developed by Yanagisawa et al. (2011) involved labeling amyloid with ^19^F-containing compounds. As biological tissues contain no ^19^F, detection of plaques can be improved by reduction in the background non-specific signal (Higuchi et al., 2005; Yanagisawa et al., 2011). Similarly novel approaches for PET ligand development have been explored. An example of this is the targeting of metal-associated Aβ aggregates with the metal chelator, clioquinol labeled with an iodine radioisotope (Roney et al., 2009; Kulkarni et al., 2010). In a parallel approach, Fodero-Tavoletti et al. (2010) used *bis*(thosemicarbazonato) Cu^II^ complexes, which have been shown to facilitate intracellular Cu delivery, coupled with positron emitting ^64^Cu as a PET tracer. As this approach does not rely on uptake of Cu by plaques and consequently does not correlate with amyloid burden, they suggest it may offer complementary information to other methods.

## Conclusion

Our understanding of the pathophysiological mechanisms of AD has been significantly enhanced through the application of neuroimaging to visualize these processes *in vivo.* At the same time, our knowledge of AD pathophysiology is helping to inform new directions for imaging approaches for studying neuropathology. With the focus shifted to earlier diagnosis at a prodromal or even asymptomatic stage of AD, current imaging methods are important to define these stages. Early diagnostic criteria will depend not only on current biomarkers combined in a multimodal approach, but also the development of novel techniques for neuroimaging that may offer more specific biomarkers of disease, intimately reflecting the underlying pathophysiology. This is also important to the efficacy and success of developing new interventions for altering the course of the disease. Ultimately, without effective interventions, the ability to diagnose AD at any stage will allow us to be little more than informed observers to an unfolding health crisis.

### Conflict of interest statement

The authors declare that the research was conducted in the absence of any commercial or financial relationships that could be construed as a potential conflict of interest.
